# A Novel Approach against *Salmonella*: A Review of Polymeric Nanoparticle Vaccines for Broilers and Layers

**DOI:** 10.3390/vaccines9091041

**Published:** 2021-09-18

**Authors:** Keila Y. Acevedo-Villanueva, Gabriel O. Akerele, Walid Ghazi Al Hakeem, Sankar Renu, Revathi Shanmugasundaram, Ramesh K. Selvaraj

**Affiliations:** 1 Department of Poultry Science, College of Agricultural and Environmental Sciences, University of Georgia, Athens, GA 30602, USA; ka46846@uga.edu (K.Y.A.-V.); goa17789@uga.edu (G.O.A.); walid.alhakeem@uga.edu (W.G.A.H.); 2 Upkara Inc., 45145 W 12 Mile Rd, Novi, MI 48377, USA; srenu@upkara.com; 3 USDA-ARS, Toxicology and Mycotoxins Research Unit, Athens, GA 30605, USA; revathi.shan@usda.gov

**Keywords:** *Salmonella*, vaccines, polymeric, nanoparticles, broilers, layers, poultry

## Abstract

This work discusses the present-day limitations of current commercial *Salmonella* vaccines for broilers and layers and explores a novel approach towards poultry vaccination using biodegradable nanoparticle vaccines against *Salmonella*. With the increasing global population and poultry production and consumption, *Salmonella* is a potential health risk for humans. The oral administration of killed or inactivated vaccines would provide a better alternative to the currently commercially available *Salmonella* vaccines for poultry. However, there are currently no commercial oral killed-vaccines against *Salmonella* for use in broilers or layers. There is a need for novel and effective interventions in the poultry industry. Polymeric nanoparticles could give way to an effective mass-administered mucosal vaccination method for *Salmonella.* The scope of this work is limited to polymeric nanoparticles against *Salmonella* for use in broilers and layers. This review is based on the information available at the time of the investigation.

## 1. Salmonella

Infection with *Salmonella* is among the zoonotic diseases of most concern in the United States [1]. Ingestion of contaminated poultry meat and poultry products is a frequent source of food poisoning in humans. In an effort to assist in the prevention of human food poisoning many researchers are focused on studying the pathogenicity of *Salmonella* and different vaccination methods against *Salmonella* in poultry.

### 1.1. A Potential Health-Risk Source for Humans

The genus *Salmonella* was named in honor of the veterinary pathologist Dr. Daniel Salmon in the 1900s [2]. *Salmonella* belongs to the Enterobacteriaceae family and is a rod-shaped, Gram-negative, facultative anaerobe, that can infect a large variety of hosts [3]. There are host-specific and non-host specific *Salmonella* serotypes that are found in poultry. *Salmonella* Gallinarum is the only serovar that is specific to avian hosts [4]. Non-host-specific *Salmonella* serotypes are a potential health risk source for humans because they can spread through the consumption of contaminated poultry products. *Salmonella* infection in humans is known as salmonellosis, and it results in “food poisoning” that can be manifested as diarrhea, fever, abdominal cramps, and vomiting. The severity of the symptoms depends on (1) the type of strain associated with the infection and (2) the age and health status of the host [5]. The symptoms are harsher among pregnant women, children, the elderly, and individuals who are immunocompromised. Although the infectious dose varies among *Salmonella* strains and host status, the infective dose for *Salmonella* can be as low as one cell [5]. Risk assessments have also reported that in a healthy individual about 1 × 10^6^ bacterial cells are needed to cause an infection [6]. However, it was reported that 27 CFU/g and 200 CFU/g of *Salmonella* enterica serovar Enteritidis (*S. enteritidis*) is enough to cause an outbreak that affects both healthy children and adults [7,8]. In individuals who use antacids, the infective dose is estimated to be around Log 3 *Salmonella* CFU [9].

Poultry is considered a major reservoir for many *Salmonella* serovars, making salmonellosis a global issue. For example, in the United States of America, approximately more than 70% of human salmonellosis cases have been linked to the consumption of contaminated chicken, chicken products, or eggs [10]. *Salmonella* enterica serovars Typhimurium (*S*. *typhimurium*) and Enteritidis are known to represent the main risk for public health. However, since 2018 there has been an increase in *Salmonella enterica* serovar Infantis (*S. infantis*) in poultry [11,12]. A recent outbreak of food poisoning linked to fresh chicken meat contaminated with *S. infantis*, resulted in the infection of 129 people from 32 states, 25 hospitalizations, and one death reported in New York [11].

In Europe, *Salmonella* is the second most-common cause of foodborne outbreaks [13], from which *S. enteritidis* and *S. infantis* have also been recently linked to frozen, raw, breaded chicken products that have caused almost 500 illnesses and one known death in 2020 [14,15].

*Salmonella* prominence has impacted the economy, producers, the food processing industry, and handling guidelines; hence, society. The economic burden of *Salmonella* spp. linked to chickens is estimated to be around $2.8 billion [16]. To reduce the cost burden that *Salmonella* brings, different control strategies are constantly implemented. *Salmonella* control strategies and interventions start from poultry production on the farm until the products get to the table of consumers. The system is aimed at prevention at the farm level, strengthening food safety standards for *Salmonella* surveillance, training food handlers on best practices in preventing *Salmonella*, and educating consumers [17]. At the farm level, there are precommensal strategies (probiotics, prebiotics, synbiotics), antipathogenic strategies (vaccines, organic acids, essential oils), exposure reduction strategies (biosecurity, water/feed quality hygiene), and animal handling practices (timing feed withdrawal before slaughter). Postharvest interventions include slaughter and carcass processing strategies, meat-processing strategies, and sampling and testing at critical control points [18,19]. Some of these strategies include (1) testing for the presence of *Salmonella* at farm sites before the slaughter period, (2) the use of chillers and peracetic acid, and (3) establishing sampling programs that will start from the live bird stage, during processing, and up to the final product. For the consumers, different programs provide guidelines to keep a “*Salmonella*-free” environment. For example, the Centers for Disease Control and Prevention give an “easy four-step” guideline based on remembering to clean, separate, cook, and chill poultry meat [20].

With the increasing consumption and demand for poultry products across the globe, *Salmonella* has become a potential health risk for humans. Therefore, a novel effective intervention is required to prevent an increase of *Salmonella* outbreaks.

### 1.2. Salmonella and Poultry

*Salmonella* primarily infects its host via the oral route; however, infection of *Salmonella* after exposure to “aerosolized *Salmonella*” has also been demonstrated in chicks [21]; suggesting that airborne transmission might occur. In poultry, *Salmonella* colonization of the gastrointestinal tract (GIT) can occur due to horizontal transmission when birds infected with *Salmonella* shed the bacteria in their feces and as a result, infect the environment and other closely housed birds [22]. Young birds are mainly susceptible to *Salmonella* colonization by horizontal transmission at the hatcheries during feeding, handling, and transportation [23]. *Salmonella* infection can also occur due to vertical transmission when *Salmonella* infects the ovaries of egg-laying hens [24]. The infected hen then passes the contamination to the eggs before the formation of the shell, which results in infected progeny. Furthermore, mobile *Salmonella* can cause infection directly by ascending from the cloaca to the oviduct [24]. Interestingly, *Salmonella* can colonize the GIT of birds without causing disease [22]; hence, poultry can be infected with up to Log 5 CFU *Salmonella* [25] and be asymptomatic carriers.

**Figure 1:**
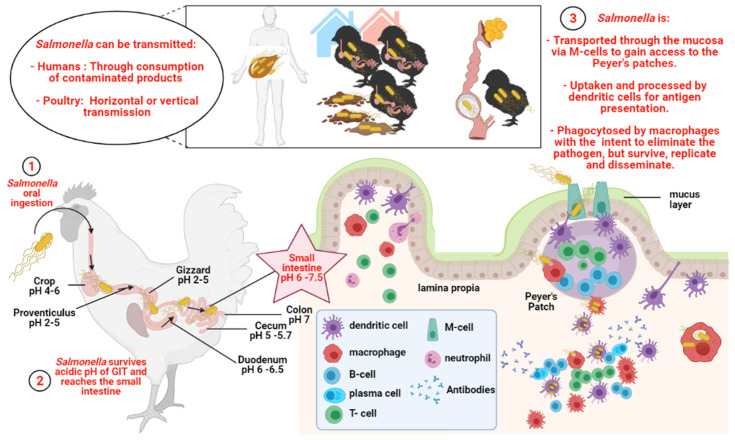
Schematic representation of *Salmonella* invasion in poultry. Created with BioRender.com (accessed on 11 September 2021).

After oral ingestion, *Salmonella* can easily thrive as an enteric pathogen due to its ability to persist in both acidic and basic environments, within a pH range of 4 to 9. Some *Salmonella* can grow at a pH as low as 3.7 [26]. In brief, upon oral ingestion, *Salmonella* will survive passage through the low-pH conditions of the gut. As it reaches the small intestine, the *Salmonella* bacterium adheres to and invades the intestinal epithelial cells [27]. In the intestinal epithelia, *Salmonella* can be transported through the mucosa, chiefly via microfold cells (M cells), to gain access to the submucosa and underlying lymphoid tissue [28]. Subsequently, macrophages within the underlying lymphoid tissue engulf *Salmonella* cells in an effort to eliminate the pathogen, yet are unable to kill them due to the ability of the bacteria to interfere with phagosome-lysosome fusion [29]. Additionally, other phagocytes, such as dendritic cells (DCs) and polymorphonuclear cells can also phagocytose *Salmonella*. *Salmonella* enterica uses many virulence factors to thrive within the host, but it specifically utilizes the type III secretion systems (T3SS) that are encoded in the *Salmonella* pathogenicity island 2 (SPI2) for its survival and intracellular replication within phagocytes [30]. Within macrophages, *Salmonella* proliferates and is eventually disseminated [31]. The typhoidal *Salmonella* serotypes can cause typhoid fever by disseminating from the intestinal mucosa [27] and invading the bloodstream and distant organs, for example, the spleen, liver, and gallbladder [31]. Non-typhoidal *Salmonella* infections, with serovars such as *S. typhimurium* and *S. enteritidis* [32], can be invasive or non-invasive [33,34]. Non-typhoidal/non-invasive serotypes usually remain localized to the GIT, causing inflammation of the mucosa and secretory diarrhea [33,35]. Figure 1 shows a schematic representation of *Salmonella* invasion in poultry. 

## 2. Immune Response against *Salmonella* in Chickens

Immune response against *Salmonella* in chickens involves the interaction of both the cellular and humoral components of the immune system. However, further research is still needed to understand their specific roles, because there are no consistent patterns between studies and there are some disagreements within the literature. Overall, *Salmonella* enterica infections in chickens have shown the involvement of different subsets of T-cells, cytokines, chemokines, and antigen-specific antibodies.

### 2.1. Innate Immunity‒Heterophils

The innate immune system is the first line of defense against pathogens. Heterophils are key players of the chicken’s innate immune system. Following an acute inflammation, the avian innate immunity is characterized by rapid influx and activation of heterophils to the intestine. Heterophils have an array of toll-like receptors (TLRs), are phagocytic, and rely on antimicrobial peptides to kill bacterial pathogens [36,37]. Chicken heterophils can phagocytose opsonized and non-opsonized *Salmonella*. It has been reported that *S. enteritidis* that was opsonized by a complement-mediated receptor had a decrease in IL-1β and IFN-γ mRNA expression, and an increase in TGF-β4 when compared to the gene expression profile of *S. enteritidis* that was opsonized by FcR (antibody) [38]. Results also showed that there were no significant differences between IL-6, IL-8, and Il-18 mRNA expressions. However, another study reported that priming heterophils with recombinant chicken IL-2 induced an increased expression of IL-8 and IL-18 for both the receptor-mediated phagocytosis of opsonized and non-opsonized *S. enteritidis* [39]. Both findings suggest that there are different signaling pathways involved in the downstream immune response of opsonized and non-opsonized phagocytosis of *Salmonella*. Following phagocytosis, avian heterophils can kill pathogens by oxidative burst, cellular degranulation, or production of extracellular matrices of DNA and histones [40]. For example, it has been reported that chicken cathelicidin-2, an antimicrobial peptide, is present in large amounts in the Type I granules of chicken heterophils; and that stimulation with *Salmonella* lipopolysaccharide triggers the release of mature cathelicidin-2 [41].

It has been reported that chicken heterophils express TLR 1, TLR 2, TLR 3, TLR 4, TLR 5, TLR 6, TLR 7, and TLR 10 [42,43]. Chicken heterophils also express TLR 15, which is a chicken-specific TLR [44]. Interestingly, a study reported that the heterophils that were isolated from fast-feathering chickens (Line A) and slow-feathering chickens (Line B), showed no differences in TLR 4 or TLR 5 mRNA expression levels, but TLR 15 was significantly upregulated in Line A heterophils before and following stimulation with *S. enteritidis* [45]. Another study demonstrated that the heterophils that were isolated from Line A had an increased ability to degranulate and produce a greater oxidative burst response when compared to the heterophils from Line B [46]. The findings indicate that the expression of TLR 15 can vary in different bird lines and that it contributes to a different response against *Salmonella* infections. Another TLR in heterophils that also varies between birds is TLR 4, which differs among breeds. For example, following in vitro stimulation with *S. enteritidis*, the heterophils from Leghorns have been shown to have the highest TLR 4 expression over the broilers and the Fayoumi line [47]. Overall, it has been reported that chicken lines that have highly functional heterophils are less susceptible to intestinal pathogens, including *S. enteritidis* [48]. These results indicate that genetic factors can significantly contribute to the heterophil TLR activity, which can determine whether a line of birds is more resistant or susceptible to *Salmonella*.

### 2.2. Cellular Immunity‒T-Helper Cells, T-Regulatory Cells, and Th 17 Cells

*Salmonella* is an intracellular bacterium; hence, cellular immunity plays a key role in *Salmonella* infections. The involvement of T-helper cells (CD4^+^) and T-cytotoxic cells (CD8^+^), and macrophage cells in avian *Salmonella* infections have been confirmed with much research over the years. It is also known that the γδ-T-cells are in greater numbers in the chicken gut, that the γδ -T cells play a crucial role in activating the adaptive immune response in the ceca and the ileum, and that a *Salmonella* infection results in an influx of γδ-T cells [49,50].

Early research has shown that chickens infected with *S. enteritidis* display changes in macrophage and lymphocyte cell populations in the ovaries and oviducts. The study reported that macrophage numbers decrease in response to *S. enteritidis* infection but return to basal levels within 21 days postinfection [51]. At day 21 postinfection, the numbers of T-cells and B- cells in the ovaries and oviducts also returned to preinfection levels. Another study reported that CD4^+^ and CD8^+^ T-cells significantly increase in the reproductive organs of *S. enteritidis* infected chickens during the first 2 weeks postinfection [52]. Other research has also reported a significant T lymphocyte increase at 2 days post a secondary *S. enteritidis* infection, for example, CD4^+^ T-cells in the spleen and CD8^+^ T-cells in the thymus [53]. Even though a specific mechanism of the role of T-cell responses in the clearance of *Salmonella* enterica has not been proven, these results suggest that CD4^+^ and CD8^+^ lymphocytes have an important role against *S. enteritidis*. Furthermore, a study also reported a decrease in the number of macrophages in the bursa of Fabricius and spleen after administering non-attenuated and attenuated *Salmonella* Typhimurium strains [54]. It was suggested that the macrophage immunosuppression that is observed during *Salmonella* infections may play a critical role in the development of the “*Salmonella* carrier status” in chickens [54], as *Salmonella* utilizes macrophages to invade the host.

Moreover, T-regulatory (T-regs) cells are known to play an essential role in *Salmonella* resistance in chickens [55]. Research has found that a persistent intestinal *S. enteritidis* infection can increase the T-regs percentage in the cecal tonsils of broilers [55]. A rise in T-regs cells results in an increase in IL-10 production that suppresses the immune response by inhibiting IL-12 production and the Th1 immune responses [56]. T-regs in the ceca promote the production of anti-inflammatory cytokines, which allow *Salmonella* to survive and remain asymptomatic in the host for long periods.

There has been little research done regarding avian Th-17 cells and *Salmonella*. A study reported that chickens challenged with *S. enteritidis* showed the expression of upregulated IL-17 in the cecum during the first week of life [57]. Newly hatched chicks responded to *S. enteritidis* with a Th1 type immune response while birds older than 10 days responded to *S. enteritidis* with a Th-17 type immune response. However, no specific role has been attributed to avian Th-17 cells against *Salmonella*.

### 2.3. Cytokines‒Th1 Proinflammatory and Th2 Anti-Inflammatory Cytokines

The mechanisms of cytokine immune response in the avian host during *Salmonella* infection is not fully understood, but in chickens, the clearance of primary infection of *Salmonella* has shown to be dominated by a Th1-type response that could occur in an organ or a time-dependent manner. It has been reported that IFN-γ is a key cytokine that initiates the proinflammatory response in the liver of Rhode Island Red chickens that were infected with *Salmonella* Typhimurium at day 7 of age [58]. A significant increase in IFN-γ expression in the liver at 7 to 14 days of infection was correlated with an increase in both CD4^+^ and CD8^+^ T-cells, which began to decline in the spleen, but increased in the ileum at 14 days postinfection. During a secondary infection, the levels of proinflammatory cytokine IFN-γ showed few changes, but the levels of IL-6 and CC macrophage inflammatory protein (CC MIP) family chemokine had a significant and rapid increase in the cecal tonsils, ileum, and intestinal tissues. For this reason, it is hypothesized that IL-6 and the CC MIP family chemokine play a major role in the recruitment of T-cells to the intestinal tissues and protective immunity against *Salmonella* infection in chickens. Furthermore, *S. enteritidis* infection in chicks can also upregulate chemokines CXCLi1 and CXCLi2, the equivalent of mammalian Il-8, which actively recruit monocytes and macrophages [59]. Overall, it has been shown that the expression of the proinflammatory cytokines IL-1β, IL-8, IL-12, IL-17, IL-18, TNF-α, and IFN-γ in combination with inducible nitric oxide synthase (iNOS) increases in the ceca of chickens infected with *Salmonella* enterica [57,60]. An increase in IL-18 and IFN-γ mRNA expression can also occur in the spleens of day-old chicks challenged with *S. enteritidis* [61].

In addition, a rapid increase in the host’s Th1 proinflammatory cytokine signals in response to *Salmonella* infection can also be used to further invade the host’s cells. *Salmonella* can take advantage of the downstream proinflammatory immune response to increase its survival rate “strategically”. The “strategic” rapid killing of the host macrophages gives way to an inflammatory environment that leads to the recruitment of more phagocytic cells to the site of the initial infection. Under this inflammation scenario, *Salmonella* can then infect more host cells, but not to kill; instead, it hides and replicates inside to avoid the host immune system [59].

Th2 cytokines dampen Th1 cytokines’ immune response and vice versa. So, while a Th1 proinflammatory response is needed to fight against a *Salmonella* infection in the chicken, a Th2 anti-inflammatory cytokine response is linked towards *Salmonella* resistance. A gene expression profile study with *S. enteritidis* reported that the Th1 activity is inhibited during the carrier-state in chicks, and instead, the majority of the genes that were differentially upregulated were linked to the Th2 network [62]. These results suggest that during the carrier-state the inflammatory response could be downregulated and that the susceptibility to *Salmonella* is associated with a Th2 bias. These findings correlate with previous research that identified that a persistent intestinal *S. enteritidis* infection increases the T-reg percentage, and thus the birds in the carrier state display an increase in the Th2 anti-inflammatory IL-10 cytokine (explained in Section 2.2).

### 2.4. Humoral Immunity‒B-Cells and Antibody Production

It has been identified that humoral immunity also has a critical role in *Salmonella* infections. *Salmonella* can target and suppress B cells and IgG production to evade and invade the host’s immune system. A recent study identified that as a strategy to evade humoral immunity *Salmonella* can specifically reduce IgG titers in serum, due to the secretion of the SiiE adhesin protein, which reduces plasma cell numbers [63]. Another study with chickens showed that the depletion of B-cell precursors and B-cells causes the intestinal shedding rate of *S. enteritidis* to increase [64]. Another study reported that after *S. enteritidis* infection, hens produce specific IgM, IgG, and IgA antibodies against *S. enteritidis* at similar levels in both serum and oviducts [51], which were in correlation with a reduction in the bacterial load in the oviducts.

However, there are also inconsistencies in the literature regarding the role of B-cells and antibody response in the clearance of *Salmonella* infection in chickens. A study with B-cell deficient chickens reported that the clearance of *Salmonella* Typhimurium primary and secondary infections in chickens is independent of B-cells or antibodies [65]. Even though the B-cell deficient birds by surgical bursectomy lacked an antibody response against *S. typhimurium*, they controlled the infection at a similar rate than that of the control, “intact”, chickens during the primary and secondary infections [65]. Results suggested that high levels of *Salmonella*-specific antibodies around the time of clearance do not imply an effective response. Interestingly, the birds that were B-cell deficient by treatment with cyclophosphamide during the first day’s posthatch were less efficient at clearing *Salmonella* from the gut—suggesting that they could have a deficiency in a non-B cell subset that did not allow them to recover from the infection. Even more interesting is that a second challenge of the birds in the surgical bursectomy group and the cyclophosphamide group showed the same rate of clearance when compared to the intact birds—suggesting that the mechanism of clearance is different between the primary and the secondary infection. Currently, it remains unknown which non-B-cell immune mechanisms aid in mediating *Salmonella* clearance.

## 3. Mucosal Vaccine Immune Response

**Figure 2:**
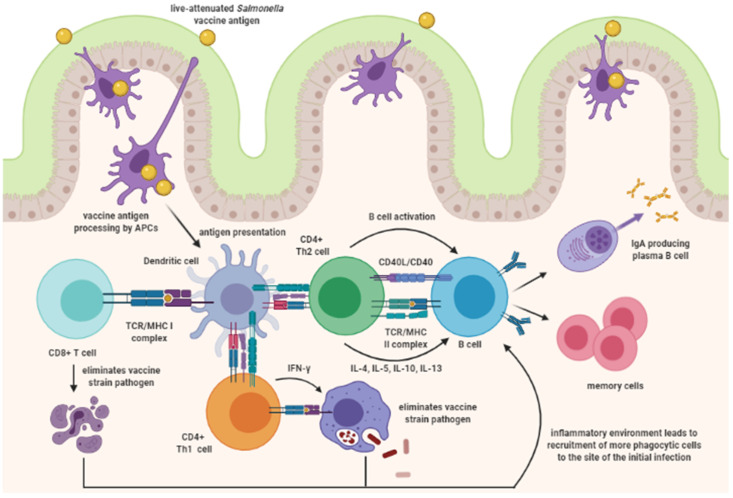
Schematic representation of the immune response induced after inoculation with a live‒attenuated *Salmonella* vaccine. Created with BioRender.com (accessed on 28 July 2021).

*Salmonella* vaccines that are administered through the oral route should induce a substantial cellular immune response that activates both Th1 and Th2 cells. The Th1 cells mediate cellular immunity by producing IFN-γ and tumor necrosis factor (TNF)-α to activate macrophages and aid other phagocytic cells, such as cells DCs, and B cells [66]. The Th2 cells mediate humoral immunity by producing cytokines such as IL-4, IL-5, IL-10, and IL-13 to mediate B-cell activation, and for antibody production [67]. Production of Secretory IgA (sIgA) aids in blocking the attachment of the bacteria, such as *Salmonella*, to the mucous layer. Figure 2 is a schematic representation of the immune response induced after inoculation with a live-attenuated *Salmonella* vaccine. Ultimately, the goal of mucosal vaccination is to generate memory cells that can immediately aid against a mucosal pathogen, like *Salmonella*.

### 3.1. Mucosal Vaccination Practicality for Poultry

Mucosal vaccination, such as nasal, oral, or ocular, is the concept of administrating a vaccine at a mucosal site, that contains overlying mucosal fluid, to induce a localized immune response in the mucosal tissues [68]. Mucosal vaccination is a critical approach to pathogens such as *Salmonella* that make their entrance to the host via mucosal tissues. *Salmonella* can be easily transferred to nearby birds by horizontal transmission. This becomes a problem in a commercial setting because the flock sizes of commercial poultry operations contain thousands of birds. For this reason, it is very important to have efficient methods of mass vaccination to prevent losses. As a vaccination method, the oral delivery route—for example, via feed, water, or oral gavage—has many benefits in the poultry industry. The oral delivery route can: (a) decrease the need for individual administration of vaccines, e.g., intramuscular injections, (b) retain the meat quality of poultry, (c) stimulate mucosal immunity, (d) be rapid, and (e) reduce bird handling, stress, and labor costs [69,70].

### 3.2. Gastrointestinal Tract Challenges

Oral immunization faces different challenges presented by the GIT. A successful immunization requires the delivery of the intact antigen of interest to the GIT, the efficient transport of the antigen across the mucosal barrier, and the successful activation of APCs. However, the main problems of oral antigen delivery are achieving successful delivery of the antigen to the GIT and ensuring the subsequent antigen uptake by APCs.

#### 3.2.1. pH of the GIT—The Physiochemical Barrier

The first challenge for oral delivery of mucosal vaccines is the acidic nature of the GIT, which can degrade or denature the antigen or drug of interest, lowering their efficiency [71]. The main function of the GIT is to digest materials that have been consumed for the absorption of nutrients. To execute its function, the GIT portrays a highly acidic environment that has a variety of pH ranges and proteolytic enzymes that are responsible for protein degradation. In chickens, the variety of pH in the GIT ranges as follows: crop 5.5 pH, proventriculus/gizzard 2.5–3.5 pH, duodenum 5.0–6.0 pH, jejunum 6.5–7.0 pH, ileum 7.0–7.5 pH, and colon 8.0 pH [72]. In this “harsh” environment, the orally administered soluble proteins are highly susceptible to protein degradation or denaturation, which compromises the vaccine delivery outcome. An orally delivered antigen needs to endure the pH ranges of 2.5–7.5 in order to successfully reach the small intestine and cross the mucosal barrier to activate APCs and ultimately create memory T-cells.

#### 3.2.2. Mucosal Barrier of the GIT—A Surprising Ally

The intestinal mucosal layer is another key component, which protects against pathogen invasion of epithelial cells. The mucosal layer coating the surface of the GI tract can act as a physical barrier to bacteria that are present in the lumen [73]. Bacteria like *Salmonella* must adhere to the mucus components to remain in the intestines [74], and key proteins in mucus prevent bacterial adhesion to surfaces [75]. In addition, the intestinal epithelial mucosa has an intrinsic negative charge. The negative charge of the mucosal layer along the GIT can be used as a strategic target to ensure the delivery of cationic antigens or drugs of interest to the intestinal epithelium [71,76,77,78]. The interaction between a highly cationic antigen and the anionic mucus layer can result in an increased mucoadhesion of the vaccine antigen that can ensure its delivery to the GIT. More details on how the cationic properties of mucus can aid in the antigen delivery of vaccine antigens are discussed in the chitosan nanoparticle section.

#### 3.2.3. Microfold Cells—The Sentinels of the Intestinal Epithelium

The epithelial monolayer underlying the mucus layer contains different cell types with diverse roles. One of the most important cell types are M cells, which sample mucosal contents and antigens and are the favored route of entry by *Salmonella* [74]. This is because the surfaces of M cells are not covered by the mucus layer [66] and they do not process the antigen [79]. Thus M cells inadvertently provide opportunities for bacterial pathogens to dock and invade [74]. M cells function to sample and transport the mucosal contents, antigens, or pathogens, from the lamina propia surface to the subepithelium. They have a unique “pocket” on the basolateral surface that allows for antigen-presenting cells (APCs) to process the engulfed molecule in a short time [79]. By acting as sentinels of the intestinal epithelium, M cells with their unique transepithelial transport, are strategic targets for potential *Salmonella* vaccines for poultry [78,80]. Underlying the M cells is the subepithelial dome (SED). The SED contains the Peyer’s patches (PPs) that have germinal centers, DCs, and macrophages [66,81]. Dendritic cells process the antigen, and they can present it to adjacent T cells, and subsequently, B cells within the follicles are stimulated to ultimately induce an antigen-specific immune response. Even though the infiltration of pathogens through M cells has been well-described, their fate at the SED remains less well-understood.

#### 3.2.4. Killed Mucosal Vaccine Antigens and Antigen Presenting Cells

Mucosal vaccines face the same challenges as mucosal pathogens. Therefore, efficient uptake, processing, and presentation of vaccine antigens by DCs is a prerequisite in shaping the nature of the adaptive immune response against a pathogen [82]. Dendritic cells are professional antigen-presenting cells that function to induce naïve T-cell activation and effector differentiation [66]. A vaccine antigen should successfully stimulate the innate immune system’s APCs to induce a protective downstream adaptive immune response to a specific pathogen [83]. However, killed or inactivated vaccine antigens are known to elicit poor cell-mediated immunity, while live vaccines elicit both cell-mediated and antibody-mediated immune responses [66]. Therefore, for killed or inactivated vaccines, adjuvants are generally required to ensure or facilitate antigen recognition and entry into mucosal sites. For the efficient antigen uptake, processing, and presentation of killed vaccine antigens by DCs, a successful adjuvant should either be a particulate to facilitate antigen uptake, be able to adhere to mucosal surfaces, or target the M cells and effectively stimulate innate responses [84], e.g., biodegradable nanoparticles [78,80].

#### 3.2.5. Killed Mucosal Vaccines and Immunoglobulin A

The hallmark of mucosal immunity is its role as a major source of IgA precursor. The local APCs and T cells are known to selectively enhance IgA responses in the GIT [68]. Secretory IgA in the mucosal surfaces has a polymeric structure that is composed of dimeric IgA, its joining chain, and a secretory component chain. Secretory IgA aids in maintaining the balance between the commensal microorganisms in the GIT and can also assist in defending the immune system from pathogens on mucosal surfaces [66]. It has been hypothesized that sIgA can also contribute to the late clearance of *Salmonella* Enteritidis from the GIT [85]. Previous research has also shown that sIgA can directly interact with flagella to inhibit the motility of *Salmonella* enterica serovar Typhimurium [86].

**Figure 3:**
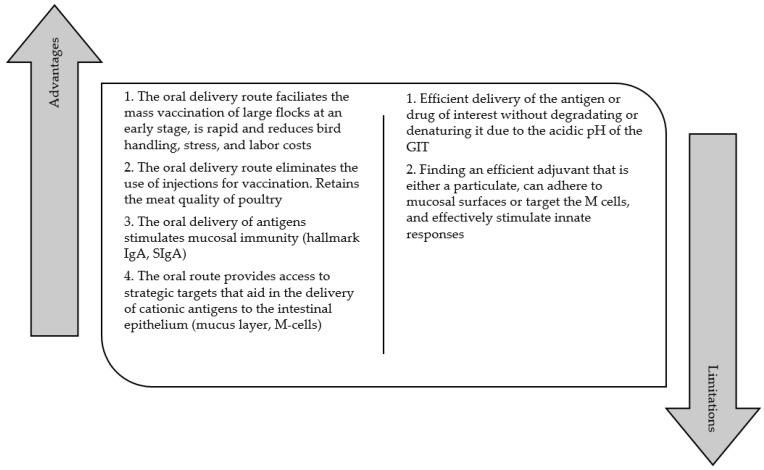
Summary of advantages and limitations of mucosal vaccination for poultry.

The route of administration for vaccines plays a crucial role in determining whether the predominant downstream response is governed by either a systemic or mucosal immune response. Systemic and mucosal immune responses are known to be segregated from one another [87]; hence, the route of administration is critical for maximizing immune responses against systemic vs. mucosal pathogens. For mucosal pathogens, like *Salmonella*, the route of administration for conventional killed vaccines for broilers is via intramuscular injections. Injected vaccines induce specific T-cell responses in the bloodstream, which result in predominant serum IgG responses; hence, they are known to induce poor mucosal immunity. Poor mucosal immunity due to the lack of sIgA in mucosal surfaces can be overcome with the oral administration of vaccines. The oral administration of vaccines through mucosal surfaces has been shown to stimulate substantial amounts of sIgA in the GIT [84,88,89]. Unfortunately, synthesizing an oral killed vaccine against *Salmonella* to substantially increase the broilers’ sIgA amounts and strengthen their mucosal immunity currently remains a challenge. More details on the challenges of mucosal vaccines against *Salmonella* for broilers are discussed in the vaccines against *Salmonella* section. Figure 3 summarizes the advantages and limitations of mucosal vaccination for poultry.

## 4. Vaccines against *Salmonella*

Currently, the United States Food and Drug Administration (FDA) does not require mandatory *Salmonella* vaccination because of the lack of data on the efficacy of current vaccines and their inability to fully eradicate *Salmonella* [88]. Instead, they strongly encourage producers to vaccinate to help reduce *Salmonella* load. Hence, poultry vaccination is aimed at preventing or minimizing the emergence of clinical disease at a farm level [89].

### 4.1. Live vs. Killed Salmonella Vaccines for Poultry

Commercially available *Salmonella* vaccines for poultry can broadly be divided into killed or inactivated vaccines and live vaccines that have major disadvantages in the poultry industry. The major concern regarding the available live *Salmonella* vaccines for poultry is the ability of the live vaccine strain to revert to its virulent form. Conversely, killed, or inactivated vaccines are preferred because they do not pose any risk of reverting the bacterial strain pathogenicity. However, the route of administration for commercially available *Salmonella* killed vaccines in poultry also has drawbacks. *Salmonella* killed vaccines for broilers and layers are administered by subcutaneous injection, and if not done properly, this can result in focal inflammatory myositis [90] and can decrease the quality of the tissue; hence, the value of the final product. Aside from decreasing the value of the final product, the individual handling of the birds for intramuscular injections is also highly impractical for commercial poultry flocks. Moreover, killed vaccines are known to elicit a lower cell-mediated immunity and shorter length of protection; hence, they are more likely to require boosters. In contrast, live vaccines can elicit both cell-mediated and humoral immune responses and rarely require a booster [66]. Table 1 summarizes the advantages and disadvantages of commercially available live and killed *Salmonella* vaccines for poultry.

### 4.2. Commercially Available Vaccines for Salmonella in Poultry

There are currently no commercial oral-killed vaccines against *Salmonella* for use in broilers or layers. Only two of the commercially available killed vaccines for *Salmonella*, POULVAC^®^ SE and POULVAC^®^ SE-ND-IB, are intended for broiler and/or layer use, and their administration route is an intramuscular injection. Likewise, two commercially available live vaccines, POULVAC^®^ ST and SALMOVAC^®^ SE are intended for broiler and/or layer use, and their administration route is either spray or oral administration. The remaining vaccines listed are intended for breeder/layer use. Table 2 summarizes the commercially available vaccines for *Salmonella* in poultry [91,92,93,94,95,96,97,98,99,100].

For a more practical scenario, the oral administration of killed or inactivated vaccines would overcome the shortcomings that are faced with the currently commercially available *Salmonella* vaccines for poultry. An oral administration approach is (1) safe for both the environment and close by birds and humans, (2) mimics a natural infection, (3) stimulates the mucosal and systemic immune responses, (4) complies with animal welfare, and (5) decreases labor cost [70].

As a novel-alternative approach, polymeric nanoparticles for *Salmonella* vaccination in poultry have been explored in recent years.

## 5. A Novel Approach to Poultry Vaccination: Nanoparticles Vaccines against *Salmonella* for Use in Broilers and Layers

Approximately ninety percent of pathogenic infections take place at mucosal surfaces [101]. Traditional *Salmonella* killed vaccines that are commercially available are administered via intramuscular or subcutaneous injections, which is impractical for commercial poultry flocks. Moreover, unlike in the administration of vaccines through injections, the oral delivery of vaccines to mucosal surfaces aids in building both systemic and mucosal immune defenses that equip the host with the necessary tools to prevent or fight infections caused by mucosal pathogens. However, there are currently no killed vaccines that are delivered through the oral route to provide dual systemic and mucosal protection for poultry flocks. Recent research has adapted the use of modern nanotechnology to improve the poultry industry [78,80,102,103,104,105,106,107,108,109]. The use of a biodegradable nanoparticle that protects the vaccines antigens from the harsh pH of the GIT and that can be “programmed” to deliver the antigens to the intestinal PPs is a new step towards innovative vaccines. As the development of new biodegradable nanoparticle vaccines against *Salmonella* for use in poultry is still in its early stages, much research still needs to be done to guarantee their commercial use. However, existing research up to date has shown promising results for the use of biodegradable nanoparticle vaccines in the poultry industry.

### 5.1. Nanoparticles

A nanoparticle is typically defined as a particle of matter that ranges between 1–100 nm in diameter [110], depending on the type of nanoparticles. Nanoparticles can be classified into different types, for example, metal nanoparticles, carbon-based nanoparticles, lipid-based nanoparticles, and polymeric nanoparticles. Polymeric nanoparticles are colloidal particles that range from 10–500 nm [111], which size allows them to cross the intestinal mucosal barrier of the GIT and facilitates their uptake by APCs [112]. Polymeric nanoparticles have been recently explored as an alternative to conventional *Salmonella* vaccines for broilers and layers.

Polymeric nanoparticles can internalize biologically active materials either through encapsulation or covalent bonding. Covalent bonds can be cleaved in response to changes in the pH [113] to control the release rate of antigens or drugs; which is favorable to overcome the challenging acidic nature of the GIT [111]. The gradual release rate of the antigen is known as the “deport effect”, which aids in prolonging the exposure of the immune system to the antigen; hence, acting by itself as a booster dose [114]. In addition, polymeric nanoparticles have an intrinsic immunomodulatory activity that permits them to act as adjuvants themselves [115].

**Figure 4:**
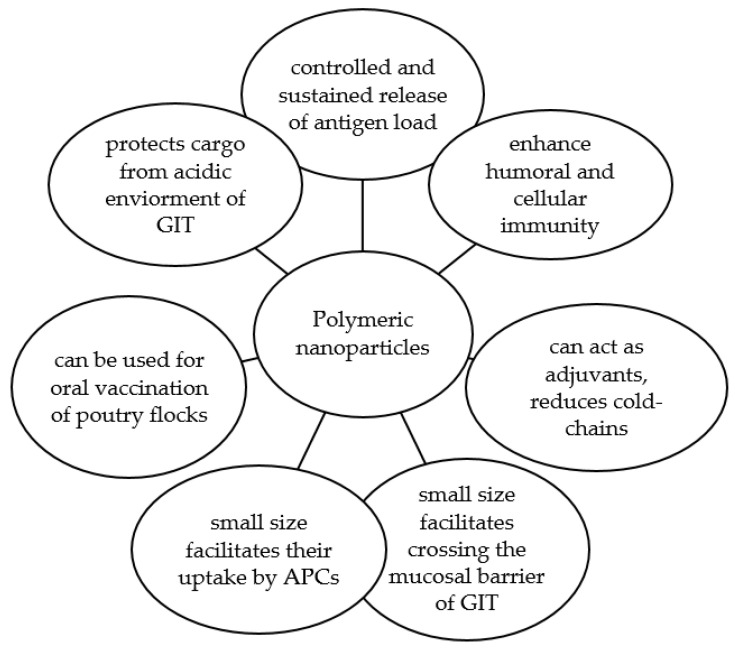
Summary of the advantages of using a polymeric nanoparticle as a delivery vehicle for poultry vaccination.

The possibility of manipulating multiple aspects of polymeric nanoparticles gives them a competitive edge as carriers for the oral delivery of vaccine antigens. As another advantage, using polymeric nanoparticle vaccines for poultry reduces the need for expensive cold chains to preserve their bioactivity. The use of polymers as carrier-delivery systems can help in improving the thermal stability of the loaded antigens at ambient shipping conditions [116]. Overall, using biodegradable nanoparticles as adjuvants and delivery vehicles for the oral vaccination of poultry flocks against *Salmonella* can overcome the shortcomings of current conventional vaccines. Figure 4 summarizes the advantages of using a polymeric nanoparticle as a delivery vehicle for poultry vaccination.

### 5.2. Nanoparticle Vaccines against Salmonella

There is a gap in the literature regarding the research of nanoparticle vaccines against *Salmonella* for use in broilers and layers. Less than 10 research articles exploring this novel approach to poultry vaccination have been published from 2018 to 2021. Table 3 provides a summary of the published literature up to date at the time of this review. Current studies explore chitosan-based and poly (methyl vinyl ether-co-maleic anhydride) (PVM/MA)-based polymeric nanoparticle vaccines for use in poultry against *Salmonella*.

### 5.3. Chitosan-Based Nanoparticles

Chitosan is a natural biodegradable copolymer that is derived from the deacetylation of chitin from the exoskeleton of crustaceans [117]. Chitosan has many commercial uses; for example, it is used in bandages to reduce bleeding, as an antibacterial agent, and it is also approved by the FDA for the safe use of foods and drugs for humans.

**Figure 5:**
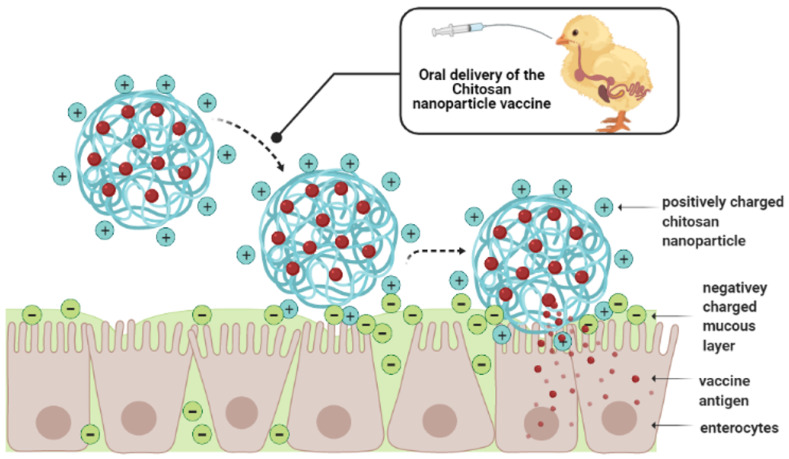
Schematic representation of the mechanism of action of chitosan-based nano-particles upon arrival at the small intestine. Created with BioRender.com (accessed on 28 July 2021).

As previously mentioned, the negative charge of the mucosal layer along the GIT can be used as a strategic target to ensure the delivery of cationic antigens, like chitosan, to the intestinal epithelium. The natural composition of chitosan plays a key role in this strategic approach. Chitosan is comprised of copolymers of glucosamine and N-acetylglucosamine. The amino and carboxyl groups in the chitosan molecule can combine with the glycoprotein in the mucus to form hydrogen bonds [118]. The ionic interaction between the cationic primary amine of chitosan and the anionic sialic acid group of mucus, results in an adhesive effect, which facilitates the targeted antigen delivery by enhanced adhesion [77,119]. Figure 5 shows a schematic representation of the mechanism of action of chitosan-based nanoparticles upon arrival at the small intestine. Overall, the increased mucoadhesive properties of chitosan give a strategic advantage over conventional *Salmonella* vaccines for poultry, which is seen in recent studies with broilers and layers.

#### *Salmonella* Chitosan Nanoparticle Vaccines in Chickens

Few studies have assessed the effects of *Salmonella* chitosan nanoparticle vaccines in chickens. Recent studies designed and demonstrated the immune response of broilers and layers to an oral chitosan nanoparticle (CNP)-based vaccine against *Salmonella* [78,102,103,104,105,106,107]. For these studies, the *Salmonella* subunit CNP vaccine was synthesized to contain *S. enteritidis* immunogenic outer membrane proteins (OMPs) and flagellin protein combined with a flagellin surface coating.

In a pioneer study, the loaded CNP vaccine was characterized and results demonstrated that the loaded chitosan nanoparticles are biocompatible in chickens, have an average size of 514 nm, and are stable when exposed to a highly acidic environment, as low as pH of 2, over a long period of time [78]. Results from multiple studies show that upon thriving in the acidic pH of the GIT, the CNP vaccines adhered to the mucosal surface and were uptaken by ileal PPs and lamina propria immune cells, while the CNP without flagellin surface coating were poorly uptaken by the PPs [78,105,109]. Results identified that the oral inoculation of loaded CNP can overcome the poor antigen delivery hurdles of mucosal vaccines. It is important to mention that the nanoparticle average size does vary from batch to batch across studies [78,105,109], which is a prevalent challenge that is faced when synthesizing nanoparticles [120]. This shows that the batch-to-batch variation is a factor that needs to be improved because it could potentially affect the vaccine’s antigen uptake by dendritic cells, which can lead to different outcomes in the immune response.

Another study evaluated the dose- and age-dependent response and efficacy of the loaded CNP vaccine in broilers [105]. Results showed that two doses of vaccine are required to induce a significant immune response. The birds that received 2 doses of 10 μg loaded CNP vaccine at 3 days and 3 weeks of age, and the birds that were inoculated twice at 3 and 4 weeks of age with 50 μg loaded CNP vaccine, had the lowest *Salmonella* loads in the ceca [105]. It was recommended that for an effective and early protection against *Salmonella*, the first dose should be given at day 3 of age or within the first week after hatching, followed by a booster after 2 weeks. Other studies have reported significance in decreasing *Salmonella* cecal load using 1000 μg loaded CNP dose at embryonic day 18 and on the day of hatching, followed by a booster at day 7 of age [102,104].

Multiple studies with broilers and layers identify that the loaded CNP can induce significantly higher antigen-specific mucosal IgA production at different time points postvaccination and postchallenge [78,102,103,104,105,106,107]. The OMPs- and flagellin-specific IgA antibody response in serum, cloacal swabs, bile, small intestine, and tracheal wash samples are significantly increased at different time-points across studies, demonstrating that the vaccine under study can provide an antigen-specific mucosal immune response that is essential against enteric pathogens. Other studies have found that CNP-immunized birds can also induce significantly higher levels of antigen-specific IgY [102,103,104], demonstrating that the CNP vaccine can potentially increase the systemic humoral immune response.

Several studies also analyzed the ability of the vaccine to induce a cell-mediated and recall-memory immune response by analyzing antigen-specific lymphocyte proliferation in PBMCs and splenocytes [78,107,109]. Immunized birds significantly enhanced rapid proliferation of OMPs and flagellin-specific lymphocytes, indicating the activation of the adaptive immune response to the CNP vaccination. The ability of the vaccine to induce both the innate and adaptative immune response following its oral inoculation demonstrates its capability of (1) blocking the primary stage of *Salmonella* infection in broilers and layers and (2) inducing an effective antigen-specific recall response.

The vaccine’s capability of regulating the immune response has also been studied by analyzing the levels of iNOS, TLRs, and Th1 and Th2 cytokines mRNA expression of immunized broilers and layers [78,102,103,104,105,106,107]. The iNOS is a key enzyme in the macrophage inflammatory response that induces the production of nitric oxide (NO), needed to eliminate pathogens like *Salmonella* [121,122]. However, when macrophages are activated during infections, NO can be produced at high levels that can result in toxic reactions against the hosts’ tissues [123,124], which could compromise the birds’ health status. The CNP vaccine has been shown to increase iNOS mRNA expression in the cecal tonsils of vaccinated broilers while not compromising the birds’ production performance status [102,104]. The CNP vaccine has also shown to significantly increase the expression of TLR-1, TLR-2, TLR-3, TLR-4, TLR-5, TLR-7, TLR-15, TLR-21, and IL-1β, IL-4, IL-10, IFN-γ, and TGF-β mRNA expression in immunized birds [78,103,104,105,107]. These results highlight that the CNP vaccine delivery system can enhance the adaptive immune response by acting as a self-adjuvant that increases the expression of different TLRs and Th1 and Th2 cytokines.

The CNP is shown to significantly decrease *Salmonella* colonization in broilers and layers when administered using either an individual oral gavage, via water, feed, or through in ovo delivery [102,103,104]. Two studies of these studies have shown that the CNP is a potential candidate for mass vaccination. A study found that the drinking water delivery of CNP can significantly reduce the challenge *Salmonella* load in the cecum by around 14 times compared to the mock-challenge load [103]. The delivery of the CNP vaccine via the feed can reduce the *Salmonella* shedding by 7 times compared to the mock-challenge and the soluble OMPs and flagellin proteins groups [103]. Another study revealed that on day 21 postchallenge, the in ovo vaccinated birds had a 0.85 Log10 CFU/g reduction of *S. enteritidis* ceca loads (*p* < 0.05) when compared to the mock-control [102]. Another study aimed to improve the efficacy of the CNP vaccine containing immunogenic OMPs and flagellin by modifying its surface coating with flagellin and mannose [106]. They observed that the oral gavage of the CNP vaccine that was surface-conjugated with both mannose and flagellin produced the greatest *S. enteritidis* reduction, by over 1 Log10 CFU/g of the cecal content. A 1 Log reduction of *Salmonella* is of biological importance as it may result in fewer contaminated carcasses that may lead to a substantial reduction in the incidence of salmonellosis in humans. Nonetheless, there is a gap in literature regarding the CNP effects on reducing *Salmonella* carcass loads; hence, future research should investigate this matter.

A different study evaluated the cross-protective effect of the CNP vaccine with a *Salmonella* enterica serovar Heidelberg (*S. heidelberg*) experimental challenge, postvaccination [104]. Results were not significant for cross-protection, but the oral gavage inoculation of the CNP vaccine numerically reduced the *S. heidelberg* loads in the liver and spleen of vaccinated broilers; suggesting that further research regarding the vaccine’s potential for cross-protection against homologous and heterologous *Salmonella* serovars needs to be carried out.

These findings show that even though the development and study of the CNP vaccine are at their early stages and ongoing, a chitosan-based nanoparticle system is suitable for the oral delivery of *Salmonella* vaccine antigens to mitigate *Salmonella* in poultry. Although progress has been made in understanding the vaccines mechanism of action and the immune responses against *Salmonella* infection post oral vaccination, further research is needed to understand the complete roles of cell and humoral-mediated immunity, because until now, different patterns have been observed between studies. For example, the efficacy in decreasing *Salmonella* colonization load in the ceca varies with the application method. Further studies should further explore how to improve consistency between batch-to-batch formulations, the vaccine’s efficacy, and performance, taking into account the differences in mass vaccination methods, further modifying the vaccine’s loaded or surface-tagged antigens, incorporating antigens from different serovars, or exploring the vaccine’s cross-protection efficacy with homologous and heterologous *Salmonella* challenges.

### 5.4. Polyanhydride-Based Nanoparticles

**Figure 6:**
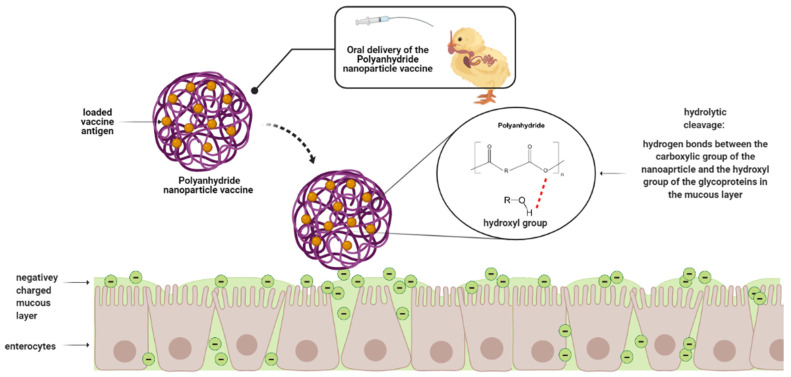
Schematic representation of the mechanism of action of polyanhydride-based nanoparticles upon arrival at the small intestine. Created with BioRender.com (accessed on 28 July 2021).

Polyanhydride is a synthetic biodegradable copolymer of non-toxic acid monomers. Polyanhydrides are characterized by anhydride bonds that link repeat units of the polymer backbone chain. The anhydride bond is formed by dehydration of diacid molecules by melt polycondensation [125]. Polyanhydrides are aliphatic, aromatic, or unsaturated [126]. Sebacic acid, adipic acid, and terephthalic acid-based polymer are the most widely studied [127]. Similar to chitosan, polyanhydrides possess mucoadhesive properties. The anhydride bonds are hydrolytically cleaved in the gut, which exposes the carboxylic acid groups that form hydrogen bonds with the hydroxyl groups of the glycoproteins in the mucus [128]. Figure 6 shows a schematic representation of the mechanism of action of polyanhydride-based nanoparticles upon arrival at the small intestine.

Polyanhydride nanoparticles are FDA-approved for use in cancer chemotherapy but have been explored recently for antigen delivery because they can act as adjuvants [129]. For example, Salman et al. (2009) was able to induce systemic Th1 and Th2 immune response using mannose-coated polyanhydride nanoparticles [130], while Tamayo et al. (2010) found that PVM/MA is an agonist of TLR -2, 4, and 5, as well as inducing a CD8^+^ T-cell response [131]. The physicochemical properties of polyanhydride such as hydrophilicity, crystallinity, and surface charge can be modified for different applications. For example, an increase in hydrophilicity by adding compounds such as polyethylene glycol results in increased biodegradability and therefore greater antigen release. In another example, polyanhydride nanoparticles synthesized by displacement of organic solvents (solvent displacement) minimize the denaturing of antigens by the solvent [129], making this method popular for vaccination studies. Ojer et al. (2013) demonstrated that polyanhydride nanoparticles loaded with different antigens and possessing a net negative charge are cytoadhesive and thus suitable for mucosal administration [132].

#### *Salmonella* Polyanhydride Nanoparticle Vaccines in Chickens

As with chitosan, there are limited studies on polyanhydride-based *Salmonella* vaccines. Two major studies conducted in 2018 and 2021 with layers are highlighted [80,108]. Both studies utilized PVM/MA loaded with *Salmonella* antigens and administered to layer birds through oral gavage [80] and drinking water [108].

In the 2018 study [80], PVM/MA nanoparticles were loaded with *S. enteritidis* flagella and OMPs proteins and were surface-coated with flagella proteins. The loaded nanoparticles had an average size of 215 nm and were stable over a range of acidic and alkaline environments for 3 h. Overall, it was reported that the synthesized nanoparticles were of the appropriate size, charge, and shape for their uptake by APCs and subsequent induction of immune responses. The oral gavage administration of the vaccine was done at 6, 9, and 12 weeks of age, with an oral *S. enteritidis* challenge at 15 weeks of age. Results indicated that the formulation was biocompatible with chickens. Furthermore, the vaccines mucoadhesion properties were demonstrated in both in vivo and ex vivo studies where the loaded and surface-tagged nanoparticles, which were delivered through the oral route, were adhered to the mucosal surface by the ileal PPs and lamina propria immune cells. Results also showed that the nanoparticles that lacked the flagellin surface coating were poorly internalized by ileal immune cells. These results highlight that the flagellin protein coating on the PVM/MA nanoparticles facilitates a better uptake of the particles than those particles without the flagellin coating.

Other results demonstrated that the loaded PVM/MA nanoparticle vaccine was immunogenic compared to unloaded proteins and non-immunized controls [80]. Immunogenicity of the loaded and surface-tagged PVM/MA nanoparticle vaccine was demonstrated in orally inoculated layer chickens and was characterized by significant enhanced levels of antigen-specific IgG in serum and bile samples, and antigen-specific IgA in bile, cloaca, and small intestine samples when compared to the mock groups [80]. The surface-tagged and loaded PVM/MA nanoparticles also significantly enhanced the CD8^+^/CD4^+^ cell ratio in spleen, increased OMPs-specific lymphocyte proliferation, and upregulated the expression of TLR-4 in chicken cecal tonsils [80]. These results highlight vaccines’ potential to induce substantial antigen-specific cellular and humoral immune responses, which are essential against intestinal pathogens like *Salmonella*.

*Salmonella* vaccines for poultry should be able to induce substantial cellular and humoral immune responses to ultimately create memory cells that will aid in preventing or fighting against a *Salmonella* infection. One of the most important parameters when assessing the efficacy of a *Salmonella* vaccine is the capability of the vaccine to decrease the *Salmonella* intestinal load. In the 2018 study [80], the layer chickens were given an experimental *S. enteritidis* challenge of 1 × 10^9^ CFU per bird at 15 weeks of age. Results showed that one out of ten birds that were immunized with soluble vaccine antigens were negative for *S. enteritidis*, and three out of nine birds that were immunized with the PVM/MA nanoparticle vaccine were positive for *S. enteritidis* by the end of the experimental period [80]. Overall, it was reported that the vaccine cleared *Salmonella* cecal colonization in 33% of vaccinated birds; however, the significance level of the CFU/g reduction in the cecal content of immunized birds was not reported in this study. Hence, the vaccine’s efficacy in clearing the *Salmonella* intestinal load in layers remains unknown. Future research with this particular PVM/MA nanoparticle vaccine should explore the vaccine’s potential to reduce *Salmonella* load in both broilers and layers.

In the 2021 study [108], PVM/MA nanoparticles were loaded with a heat extract (HE) fraction of the cell surface of *S. enteritidis* strain 449 (NP-HE). The loaded NP-HE had an average size of 430 nm, which is optimal for DCs uptake. The loaded NP-HE nanoparticle also demonstrated the capability to circumvent the current oral-antigen delivery problems. Results demonstrated that the nanoparticle sustained a high stability in tap water (pH 6.9) and acidic and basic pH for up to two hours [108]. The vaccine displays two very critical traits for the efficient delivery of vaccine’s antigens to the GIT; however, this study did not evaluate the mucoadhesion mechanism of the NP-HE nanoparticle vaccine upon oral administration to chickens. Future research should take into account the evaluation of the NP-HE’s fate in the small intestine of orally immunized chickens to better understand the vaccine’s mechanism of action upon arrival to the small intestine.

For the same 2021 study, hens were immunized by oral gavage with two doses of NP-HE at 6 and 9 weeks of age and orally challenged with *S. enteritidis* strains [108]. Bacteriological analysis of cloacal swabs, cecum, liver, and spleen were done to assess the percentage of *Salmonella*-positive samples in immunized hens. Cloacal swab samples showed a significant reduction in the excretion of *S. enteritidis* LA5. It was reported that the mechanism by which the oral vaccination of NP-HE reduced the *S. enteritidis* was unknown, but it appeared to be dose-dependent. Previous works in mice with the NP-HE identified that the vaccine’s mechanism is chiefly promoting an early proinflammatory Th1 cell response. A late Th2 response, with an increase in serum IgG1 and IgG2a, was also identified [133]. However, IgG1 and IgG2a alleles are expressed in BALB/c mice; hence, further studies are necessary to correlate the mechanism of action observed in the previous studies with hens. Although no statistical significance was observed, results also demonstrated that hens that were immunized with NP-HE and were positive for *S. enteritidis* cultures had reduced *Salmonella* percentages of internal organs two weeks after infection [108]. Altogether, these results suggest that the NP-HE nanoparticles have potential as a *Salmonella* vaccine. Future research with the NP-HE vaccine should also consider modifying the vaccine formulation to further exploit the vaccine’s potential to decrease *Salmonella* colonization in multiple internal organs. Future works should also evaluate the vaccine’s proper dosage, mechanisms of action, and should further study *Salmonella* quantification per bird.

Table 4 provides a summary of recent findings regarding polymeric nanoparticle systems for the oral delivery of *Salmonella* antigens in poultry.

## 6. Conclusions and Recommendations: Future of Polymeric Nanoparticles in the Broiler Industry

Nanoparticles have been studied in human medicine [134], cosmetics [135], and even in the food industry [136] due to their stability, immunomodulatory traits, and the flexibility of modifying their surface traits. The combination of both polymeric materials and nanoparticles gives many advantages over conventional vaccines and other types of nanoparticles, e.g., (1) they are non-toxic, biocompatible, and biodegradable, (2) they are simple to synthesize and are required in fewer quantities during preparation, (3) they are resistant to enzymatic degradation, and (4) they have higher stability, and facilitate sustained antigen or drug release [136].

It is important to monitor the materials that are used to synthesize the nanoparticles that are given to food animals because materials, such as some metals, may result in the bodily accumulation of toxins that could impact the consumers [137]. However, other materials, such as biodegradable polymers, may decrease the possibility of human consumption of residual nanoparticles through food animals. Instead, biodegradable polymeric nanoparticles degrade into small molecules, release their cargo, and should be easily cleared by the body [138]. Moreover, although biodegradable nanoparticles are still not commercially licensed for use in poultry, two common polymers, chitosan and PVM/MA, have already been approved by the FDA for clinical use [139]; which favors their approval for use in poultry. Nevertheless, regardless of the material used to synthesize the nanoparticles, all licensed nanoparticles should be studied thoroughly for biocompatibility and the safety of the host and the consumer.

Uniformity of particle size is one of the most important traits that determines the fate of nanoparticles’ internalization in immune cells. Nevertheless, polymeric nanoparticles still face a big challenge. In a laboratory setting, it is difficult to synthesize particles that remain homogeneous in shape and size, which is why they are given a range with an average size per batch. The design of well-defined nanoparticles remains a challenge as it often leads to inconsistencies, batch-to-batch variations, that can result in a critical issue when it comes to scaling up their synthesis from the lab to an industrial scale [120]. The lack of consistency could result in variations of the proposed mechanism of action for each nanoparticle, which can slightly change the vaccination outcome. Other challenges can include the relatively high cost of particle production, the low drug encapsulation efficiency, the potential problem of high initial burst release or incomplete antigen release, and the lack of standardized test protocols for each unique material as well as reference particles for validation [140]. To fill these knowledge gaps, much research is still necessary to ensure a consistent vaccination outcome, along with the vaccine’s efficacy and the hosts’ safety.

**Figure 7:**
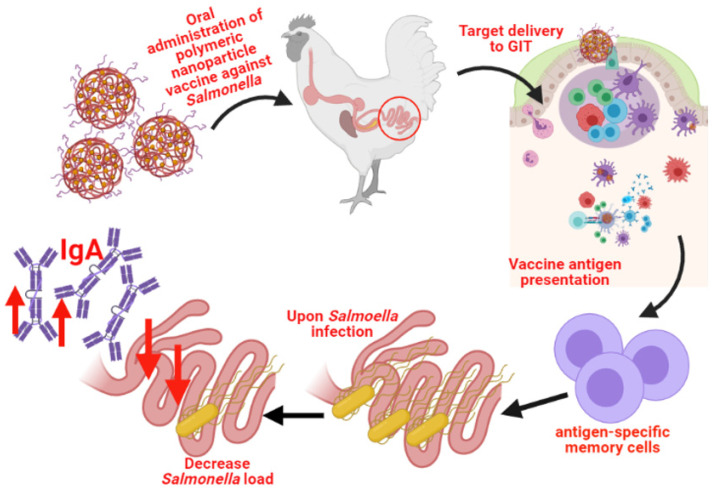
Schematic representation of the immune response upon the oral administration of biodegradable polymeric nanoparticles vaccine against *Salmonella*. Created with BioRender.com (accessed on 11 September 2021).

In terms of the use of biodegradable nanoparticles for poultry, much progress is still needed to commercialize their use. Evidently, there are still many challenges to overtake with biodegradable nanomaterials, but they hold much potential as nanocarrier delivery systems. It is evident that the use of polymeric nanoparticle vaccines for poultry is in a premature stage, but they have shown favorable results for the oral delivery of antigens against pathogens like *Salmonella*. Polymeric nanoparticle vaccines have shown to be safe for broilers and layers, capable of eliciting a substantial and antigen-specific cellular and humoral immune response in broilers and layers, and capable of reducing the intestinal *Salmonella* load in broilers and layers (Figure 7). Future research should explore (a) altering the vaccine’s compositions with proteins from different, yet prevalent, *Salmonella* serovars or different immunogenic adjuvants, (b) further exploring the vaccine’s optimal age and dose, (c) further studying the vaccine’s potential for different mass vaccination methods and possible different outcomes, (d) evaluating the vaccine’s capability to decrease *Salmonella* colonization, and can even consider (e) exploring the T-cell subsets that are involved in the immune response against *Salmonella* upon the administration of the vaccine to better understand its mechanism of action.

In the future, with increasing research in the poultry field, biodegradable nanoparticles could be commercialized as a safe and efficient antigen delivery system for the oral vaccination of birds. Polymeric nanoparticles could give way to an effective mass-administered mucosal vaccination method for prominent foodborne pathogens like *Salmonella*.

## Figures and Tables

**Table 1:** Summary of advantages and disadvantages of commercially available live and killed *Salmonella* vaccines for poultry.

Live Vaccines	Killed or Inactivated Vaccines
Live vaccine strain can revert to its virulent form and spread to the environment and to humans	Vaccine antigens do not revert to virulence. No multiplication after administration
Live vaccine strain can interfere with the salmonellosis monitoring programs	No danger of vaccine contamination
Known to elicit both cell-mediated and humoral immune responses	Known to elicit a lower cell-mediated immunity
Adjuvants in live vaccines are not common	Adjuvants in killed vaccines are often needed
Known to elicit both cell-mediated and humoral immune responses and rarely require a booster	Known to elicit a shorter length of protection, so they are more likely to require boosters to create long-term immunity
Vaccines for breeders, broilers, and layers can be administered by spray or via feed or water	Vaccines for broilers and layers are administered intramuscularly, which can decrease the quality of the tissue, hence the value of the final product

**Table 2:** Summary of the commercially available vaccines for *Salmonella* in poultry.

Company/Vaccine	Live	Killed	Bird	Administration Route
Zoetis/POULVAC^®^ ST	X		Broilers/layers	Spray
Zoetis/POULVAC^®^ SE		X	Broilers/layers	Injection
Zoetis/POULVAC^®^ SE-ND-IB		X	Broilers/layers	Injection
IDT Bio/SALMOVAC^®^ SE	X		Broilers/layers	Oral
IDT Bio/ZOOSALORAL H	X		Breeders/layers	Oral
CEVA/LAYERMUNE^®^ SE		X	Breeders/layers	Injection
CEVA/CORYMUNE ^®^ RANGE		X	Breeders/layers	Injection
ELANCO/AviPro^®^ Megan^®^ Vac 1	X		* Young chickens	Spray
ELANCO/AviPro^®^ Megan^®^ Egg	X		Layers/turkeys	Spray
ELANCO/AviPro^®^ 329 ND-IB2-SE4		X	Breeders/layers	Injection

* Young chickens is defined as “one day of age”.

**Table 3:** Updated summary of the published literature regarding nanoparticle vaccines against *Salmonella* for use in broilers and layers.

Publication	Journal	Year	Citation
Protection Conferred by Drinking Water Administration of a Nanoparticle-Based Vaccine against *Salmonella* Enteritidis in Hens	Vaccines	2021	[108]
Efficacy of a Nanoparticle Vaccine Administered In Ovo Against *Salmonella* in Broilers	PLOS ONE	2021	[102]
Chitosan-Adjuvanted *Salmonella* Subunit Nanoparticle Vaccine for Poultry Delivered Through Drinking Water and Feed	Carbohydrate Polymers	2020	[103]
Efficacy of Chitosan-Based Nanoparticle Vaccine Administered to Broiler Birds Challenged with *Salmonella*	PLOS ONE	2020	[104]
Immune Response to *Salmonella* Enteritidis Infection in Broilers Immunized Orally With Chitosan-Based *Salmonella* Subunit Nanoparticle Vaccine	Frontiers in Immunology	2020	[105]
Oral Deliverable Mucoadhesive Chitosan-*Salmonella* Subunit Nanovaccine for Layer Chickens	International Journal of Nanomedicine	2020	[78]
Mannose-Modified Chitosan-Nanoparticle-Based *Salmonella* Subunit Oral Vaccine-Induced Immune Response and Efficacy in a Challenge Trial in Broilers	Vaccines	2020	[106]
Temporal Dynamics of Innate and Adaptive Immune Responses in Broiler Birds to Oral-Delivered Chitosan Nanoparticle-Based *Salmonella* Subunit Antigens	Veterinary Immunology and Immunopathology	2020	[107]
Surface Engineered Polyanhydride-Based Oral *Salmonella* Subunit Nanovaccine for Poultry	International Journal of Nanomedicine	2018	[80]

**Table 4:** Summary of recent findings regarding polymeric nanoparticle systems for the oral delivery of *Salmonella* antigens in poultry.

Antigen Delivery System	Findings
A *Salmonella* subunit Chitosan nanoparticle vaccine synthesized to contain *S. enteritidis* OMPs and flagellin protein combined with a flagellin surface coating	Biocompatible with chickens, average size optimal for DCs uptake, and stable at highly acidic pH environment over a long period of time
	Can adhere to mucosal surface and are uptaken by ileal PPs and lamina propria immune cells
	Can induce significantly higher antigen specific mucosal IgA production
	Have also shown to significantly increased levels of antigen-specific IgY
	Can significantly enhance the rapid proliferation of OMPs and flagellin-specific lymphocytes
	Can increase significant levels of iNOS, TLR-1, TLR-2, TLR-3, TLR-4, TLR-5, TLR-7, TLR-15, TLR-21 and IL-1β, IL-4, IL-10, IFN-γ, and TGF-β mRNA expression in immunized birds
	Can significantly decrease *Salmonella* colonization in broilers and layers when administered using either an individual oral gavage, via water, feed, or through in ovo delivery
	Numerically reduced the *S. heidelberg* loads in the liver and spleen of vaccinated broilers
	Mannose modification of the CNP can reduce the *S. enteritidis* cecal load
A *Salmonella* subunit PVM/MA nanoparticle vaccine synthesized to contain *S. enteritidis* OMPs and flagellin protein combined with a flagellin surface coating	Biocompatible with chickens
	Average size optimal for DCs uptake
	Stable over a range of acidic and alkaline environments for 3 h
	Mucoadhesive and immunogenic compared to unloaded proteins and non-immunized controls
	Enhanced levels of mucosal IgA, TLR-4 and CD8^+^/CD4^+^ ratio in the cecal tonsils of immunized birds
	Cecal colonization by a homologous challenge was reduced in 33% of vaccinated birds
A *Salmonella* PVM/MA nanoparticle vaccine that is synthesized to contain a heat extract fraction of the cell surface of *S. enteritidis*	Biocompatible with chickens
	Average size optimal for DCs uptake
	High stability in tap water and acidic and basic pH
	Can significantly reduce the excretion of *S. enteritidis*
	Numerically reduced the percentages of *S. enteritidis* in cecum, liver, and spleen of the immunized hens
	Possible mechanism is chiefly promoting an early proinflammatory Th1 cell response and late anti-inflammatory Th2 response

## Data Availability

Not applicable.
